# The Long Non-Coding RNA NR3C2-8:1 Promotes p53-Mediated Apoptosis through the miR-129-5p/USP10 Axis in Amyotrophic Lateral Sclerosis

**DOI:** 10.1007/s12035-024-04059-x

**Published:** 2024-02-23

**Authors:** Dejiang Pang, Yujiao Yu, Bi Zhao, Jingxuan Huang, Yiyuan Cui, Tengfei Li, Chunyu Li, Huifang Shang

**Affiliations:** 1 Department of Neurology, Laboratory of Neurodegenerative Disorders, National Clinical Research Center for Geriatrics, West China Hospital, Sichuan University, No.37, Guoxue Lane, Chengdu, Sichuan 610041 China; 2 Department of Neurosurgery, West China Hospital, Sichuan University, No.37, Guoxue Lane, Chengdu, Sichuan 610041 China

## Abstract

Motor neuron degeneration in amyotrophic lateral sclerosis (ALS) is a form of apoptosis, but the mechanisms underlying this neuronal cell death remain unclear. Numerous studies demonstrate abnormally elevated and active p53 in the central nervous system of ALS patients. Activation of p53-regulated pro-apoptotic signaling pathways may trigger motor neuron death. We previously reported decreased expression of the long non-coding RNA NR3C2-8:1 (Lnc-NR3C) in leukocytes of ALS patients. Here, we show lnc-NR3C promotes p53-mediated cell death in ALS by upregulating USP10 and promoting lnc-NR3C-triggered p53 activation, resulting in cell death. Conversely, lnc-NR3C knockdown inhibited USP10-triggered p53 activation, thereby protecting cells against oxidative stress. As a competitive endogenous RNA, lnc-NR3C competitively binds miR-129-5p, regulating the usp10/p53 axis. Elucidating the link between Lnc-NR3C and the USP10/p53 axis in an ALS cell model reveals a role for long non-coding RNAs in activating apoptosis. This provides new therapeutic opportunities in ALS.

**Supplementary Information:**

The online version contains supplementary material available at 10.1007/s12035-024-04059-x.

## Introduction

Amyotrophic lateral sclerosis (ALS) is a fatal neurodegenerative disorder characterized by progressive muscle weakness, atrophy, paralysis, and typically resulting in death within three to five years of clinical onset [1, 2]. The defining neuropathology of ALS is the primary degeneration of upper and lower motor neurons, a disease with no precisely identified causes. Though two drugs are approved for treatment,there is currently no cure for ALS [3]. The development of various drugs for treating ALS is currently underway, but none are available with a definitive therapeutic effect. In order to improve ALS treatments, novel therapeutic strategies based on a better understanding of the disease’s pathogenesis are needed. Many pathogenic mechanisms for ALS have been implicated in the degeneration of motor neurons.An aberrant programmed cell death (PCD) or apoptosis mechanism significantly contributes to motor neuron degeneration in ALS [4]. This mechanism, or apoptosis, is governed by various interacting pathways culminating in controlled cell death [5].

Prominent in the apoptosis process is the tumor suppressor protein p53 - also referred to as “TP53” - which is produced by the tumor suppressor gene p53 located on human chromosome 17 (17p13.1) [6]. The functions of p53 gene have been comprehensively studied, whose major function is regulating cell fate after cellular stress,especially its capacity to activate apoptosis. As a transcription factor, p53 promotes apoptosis through transcription-dependent and independent mechanisms, ensuring an efficient execution of the cell death program [7]. p53 targets numerous pro-apoptotic proteins, including BAX, PUMA and AIP1 among others [8–11]. Additionally, p53 activity is meticulously regulated by posttranslational modifications to prevent over-activation leading to unnecessary apoptosis, such as phosphorylation, acetylation, and ubiquitination.The ubiquitination of p53 induces p53 nuclear export and degradation. In the presence of E3 ubiquitin ligases, p53 is ubiquitinated and degraded, and deubiquitinases (DUBs) may facilitate the reubiquitination of p53 and stabilize its levels [12–14]. Some E3 ubiquitin ligases have been shown to regulate p53 stability, such as MDM2, COP1, ARF-BP1, Pirh2 and MSL2. The ubiquitin-specific protease USP10 and HAUSP have been shown to deubiquitinate p53.

About 40 years ago, p53 was discovered, which is one of the most extensively studied genes and has previously been implicated in neurodegeneration [15]. Rising evidence suggests a substantial link between p53 and ALS. A significant increase in p53 levels and activity was detected in postmortem CNS tissues of patients with ALS [16], while another study reported substantial upregulation of p53 signaling in both induced pluripotent stem cell-derived motor neurons (iPSMNs) and post-mortem spinal cord in ALS [17]. In ALS, expression of mutant SOD1 protein (G93A) or C9orf72 repeat expansions induces DNA damage and triggers the apoptotic response by activating p53 [18–21]. In addition, a recent study suggested that p53 plays a role in protein quality control, regulating clearance of mutant SOD1 and C9orf72 DPRs [22].

Moreover, in a C9orf72 ALS mouse model, p53 ablation notably rescued neurons from degeneration and prolonged the lifespan of the mouse [23]. These findings indicate that p53 could be a promising target for ALS therapy.

Long non-coding RNA (lncRNA) is a class of RNA molecules whose transcripts are longer than 200 nucleotides, that lacks a significant open reading frame (ORF) encoding a protein [24]. Recent researches have increasingly highlighted the role of lncRNA in various physiological and pathological processes [25–27], especially lncRNA has been given more particular attention in the cancer research. As one of the most abundant ncRNA classes, lncRNAs are derived from different locations in the genome for transcription and are highly expressed in the central nervous system (CNS) [28, 29]. The role of lncRNAs has been validated in brain development, neuronal function, maintenance, and differentiation. In the meantime, lncRNAs are increasingly recognized as essential factors in the pathogenesis of degenerative diseases of the central nervous system. For instance, the lncRNA-NEAT1_2 is proposed to function as a scaffold for RNAs and RNA-binding proteins in ALS motor neurons [30, 31]. This interaction potentially modulates the function of ALS-associated RNA-binding proteins during the disease’s early phase. In a previous investigation, we identified several lncRNAs, including lncRNA NR3C2-8:1 (lnc-NR3C), with decreased levels in ALS peripheral blood leukocytes [32]. However, the roles and detailed mechanisms of these lncRNAs in ALS remain elusive.

In the present study, we explored the possible role of lnc-NR3C in ALS progression and its underlying mechanism. Our findings suggest that lnc-NR3C contributes to ALS progression by promoting apoptosis. Functioning as a competing endogenous RNA (ceRNA) [33], lnc-NR3C binds competitively to microRNA-129-5p, thereby regulating USP10-triggered p53 activation. Consequently, diminished lnc-NR3C levels suppress p53 expression, resulting in improved cell survival. These results offer novel insights into ALS’s disease mechanism and introduce a potential therapeutic target.

## Results

### Expression and Localization of lnc-NR3C in the CNS

**Fig. 1:**
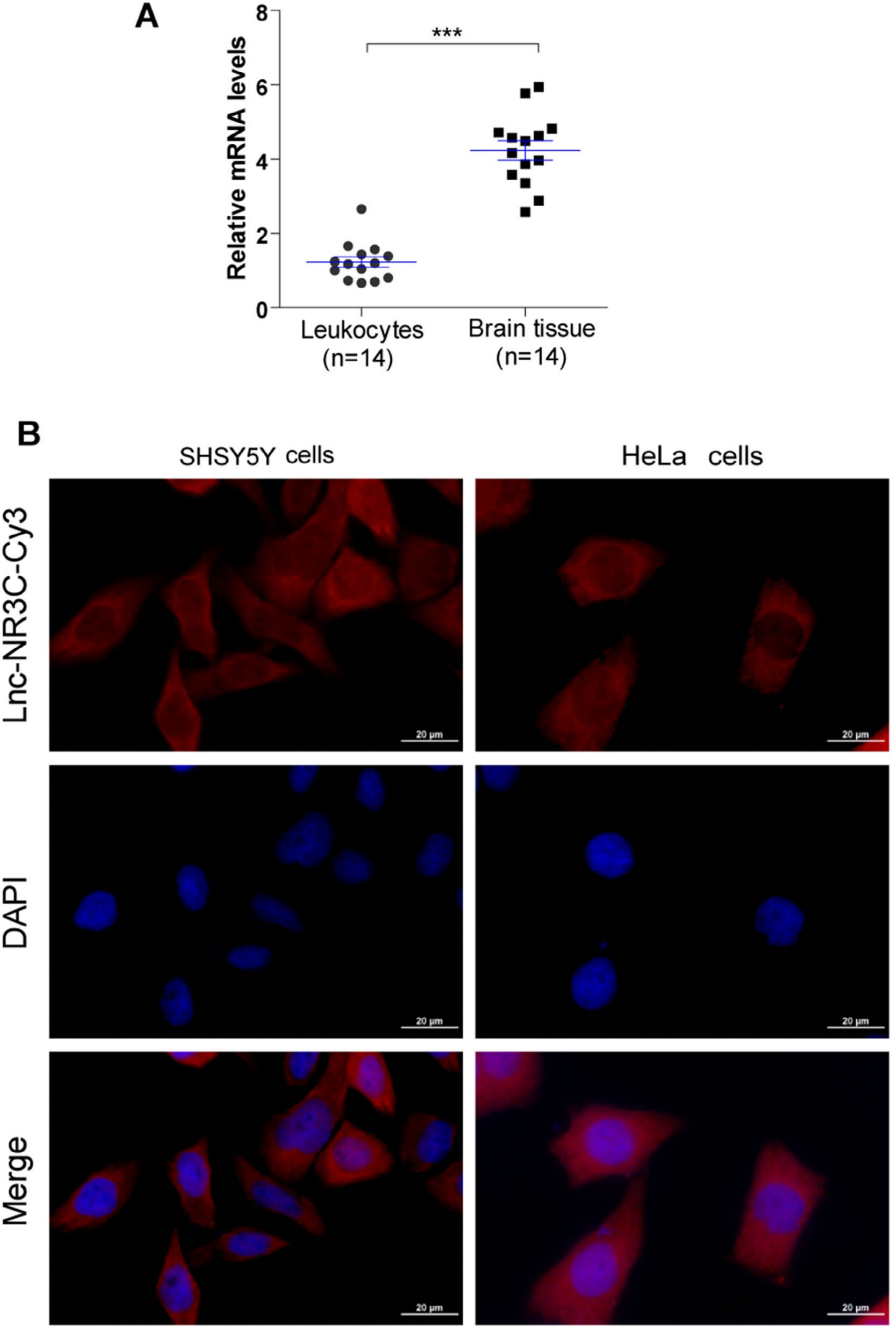
LncRNA-NR3C is expressed in the CNS and mainly localized in the cell cytoplasm. (A). lncRNA-NR3C expression in human peripheral blood leukocytes and brain tissues (peritumor tissue of glioma patients) was analysed via qRT–PCR. (***p < 0.001). (B). Localization of lncRNA-NR3C by RNA-FISH in SHSY5Y and HeLa cells. Nuclei are stained blue (DAPI), and lncRNA-NR3C is stained red. Scale bars represent 20 μm

ALS is a central nervous system disease, so as the first step towards understanding the role of lnc-NR3C, we initially investigated its expression in normal brain tissue, utilizing tissue samples obtained from the peritumoral region of glioma patients via reverse transcription quantitative PCR (RT-qPCR). Our findings revealed that lnc-NR3C is present in normal human brain tissue, exhibiting a higher expression level compared to peripheral blood leukocytes (Fig. 1A). The lnc-NR3C transcript was not conserved, and there was no expression in mouse tissues or cells. Subsequently, fluorescence in situ hybridization studies confirmed the primary localization of lnc-NR3C to the cytoplasm of HeLa and SH-SY5Y cells (Fig. 1B). These results underscore the significance of lnc-NR3C expression and localization within the CNS, providing crucial insights for understanding its function. (Table 1 should be show in supplementary information).

### Lnc-NR3C’s Role in Cell Survival and Response to Oxidative Stress

**Fig. 2:**
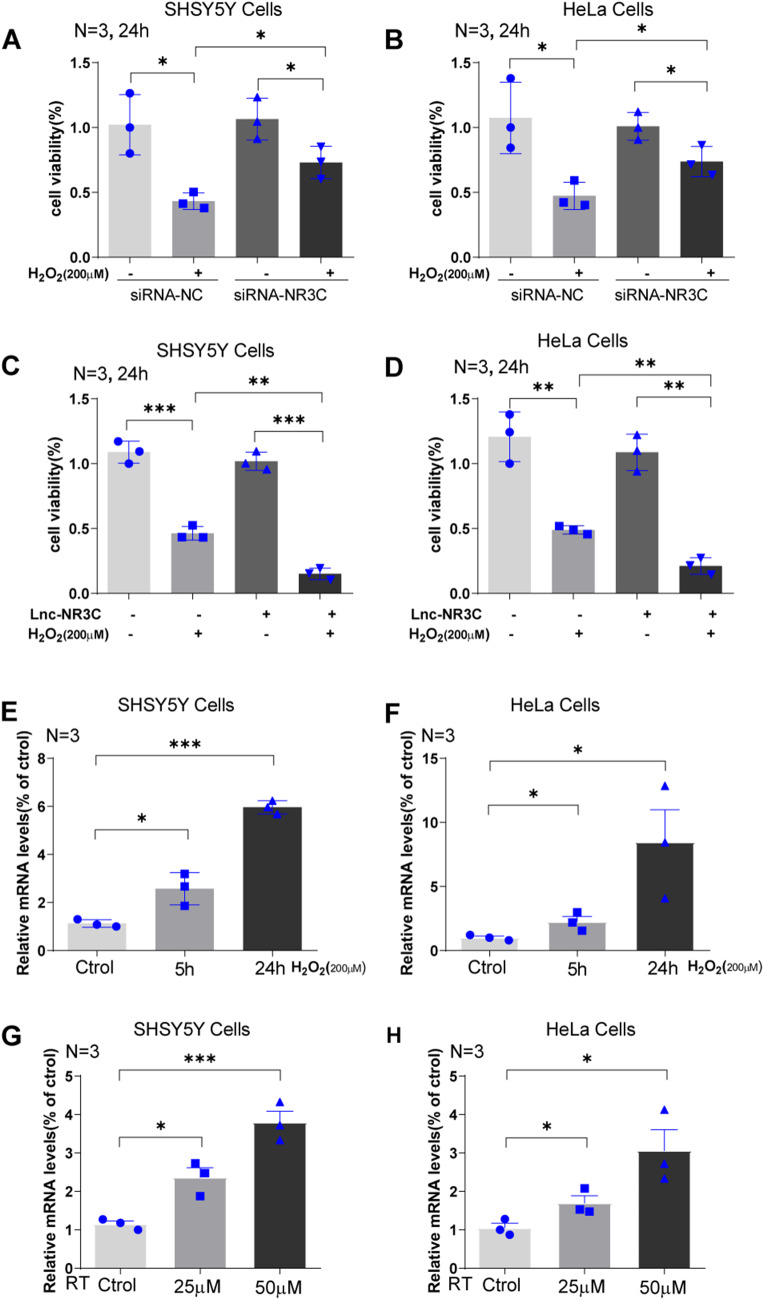
LncRNA-NR3C is involved in cell survival and induced by oxidative stress. (A-B). The cell viability of SHSY5Y cells (A) and HeLa cells (B) with lnc-NR3C knockdown were examined under H_2_O_2_ (200µM,24 h) treatment by the CCK-8 assay.(*p < 0.05). (C-D).The cell viability of SHSY5Y cells (C) and HeLa cells (D) with lnc-NR3C overexpression were examined under H_2_O_2_ (200µM,24 h) treatment by the CCK-8 assay. (**p < 0.01,***p < 0.001). (E-F).Lnc-NR3C is induced by oxidative stress evoked by H_2_O_2_ (200µM,5 and 24 h) in SHSY5Y (E) and HeLa cells (F). (*p < 0.05,***p < 0.001). (G-H).Lnc-NR3C is induced by oxidative stress evoked by rotenone (RT,25µM and 50µM,24 h) in SHSY5Y (G) and HeLa cells (H). (*p < 0.05,***p < 0.001)

Building upon our previous work, which identified a decrease in lnc-NR3C in ALS peripheral blood leukocytes, we evaluated the survival of lnc-NR3C knockdown cells under stress conditions using a CCK-8 assay. We observed a significant increase in cell survival following oxidative stress in lnc-NR3C knockdown cells compared to control siRNA-transfected cells (Fig. 2A-B). In contrast, lnc-NR3C overexpressing cells showed a significant decrease in cell survival under similar conditions (Fig. 2C-D). This data suggests that lnc-NR3C plays a role in cell survival. Furthermore, we found that lnc-NR3C RNA levels increased in association with oxidative stress induced by H_2_O_2_ (Fig. 2E-F) and rotenone (RT) (Fig. 2G-H), suggesting that lnc-NR3C may be upregulated by oxidative stress.

### Lnc-NR3C’s Promotion of Apoptosis

**Fig. 3:**
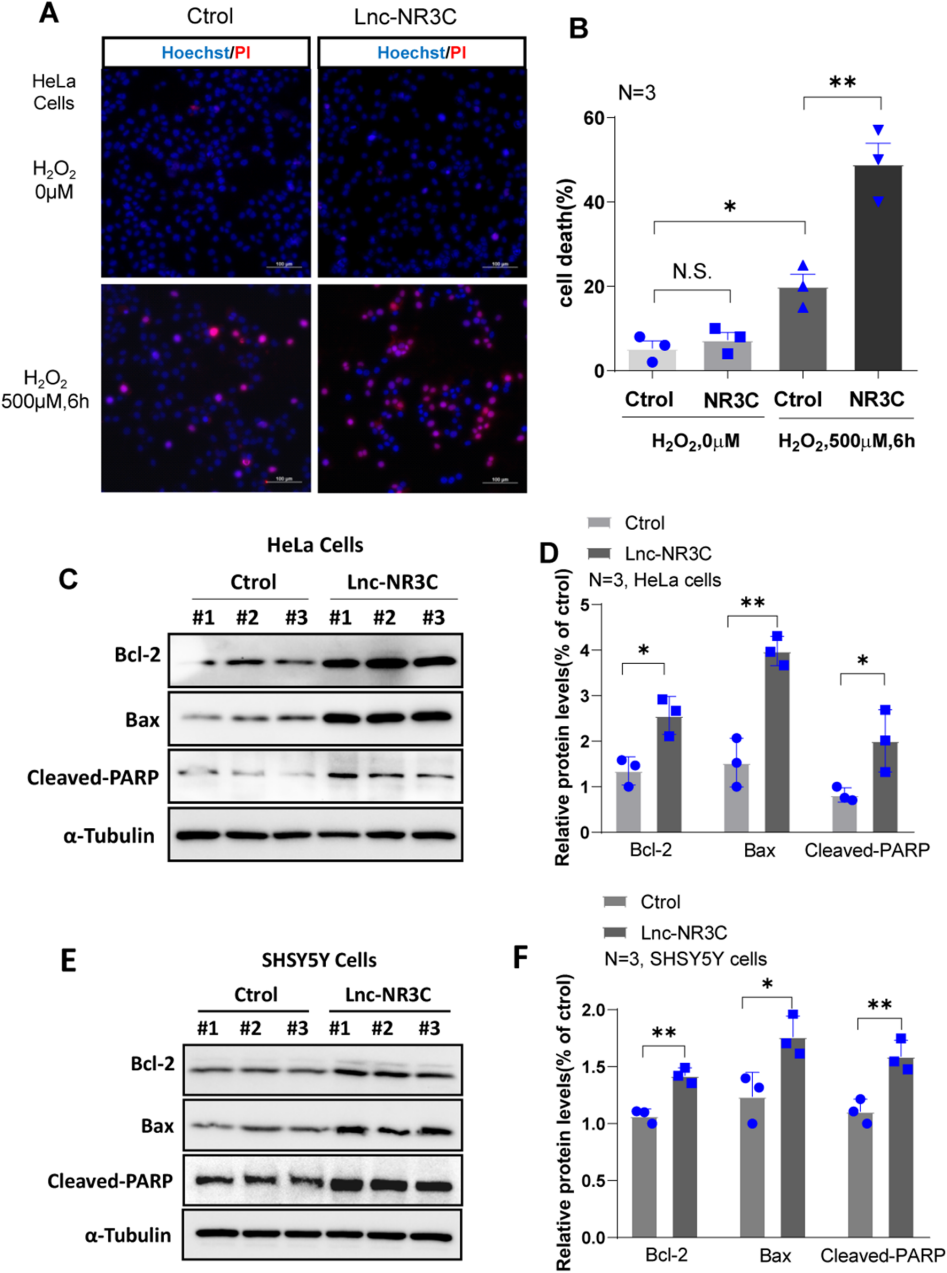
Lnc-NR3C promotes apoptosis. (A-B) After overexpressing lnc-NR3C cell apoptosis were assessed by Hochest/PI staining in the presence of H_2_O_2_(500µM,6 h).Cells were costained with Hoechst (blue), and PI (red), and visualized by fluorescence microscope (A).The total cell death is statistically analysed(B). (*p < 0.05,**p < 0.01, N.S. no statistical significance). (C-F)Western blot analysis of apoptosis-related proteins Bcl-2, Bax and Cleaved -PARP1 in human HeLa(C-D) and SH-SY5Y cells(E-F) with overexpressing lnc-NR3C.Quantification and statistical analysis of three independent experiments in HeLa(D) and SHSY5Y cells(F).(*p < 0.05,**p < 0.01,***p < 0.001)

Given lnc-NR3C’s stress-responsive regulation, we hypothesized that it might play an anti-stress role in cells. By overexpressing lnc-NR3C in HeLa cells, and using Hochest/PI staining to assess apoptosis in the presence of H_2_O_2_, we found that lnc-NR3C overexpression significantly increased cell death (Fig. 3A-B). We subsequently observed an increase in the expression of apoptosis-related proteins, including anti-apoptotic protein Bcl-2, pro-apoptotic protein Bax, and Cleaved-PARP1 (Fig. 3C-F), following lnc-NR3C overexpression. Collectively, these findings suggest that lnc-NR3C promotes apoptosis.

### Lnc-NR3C Overexpression Enhances p53 Protein Stability

**Fig. 4:**
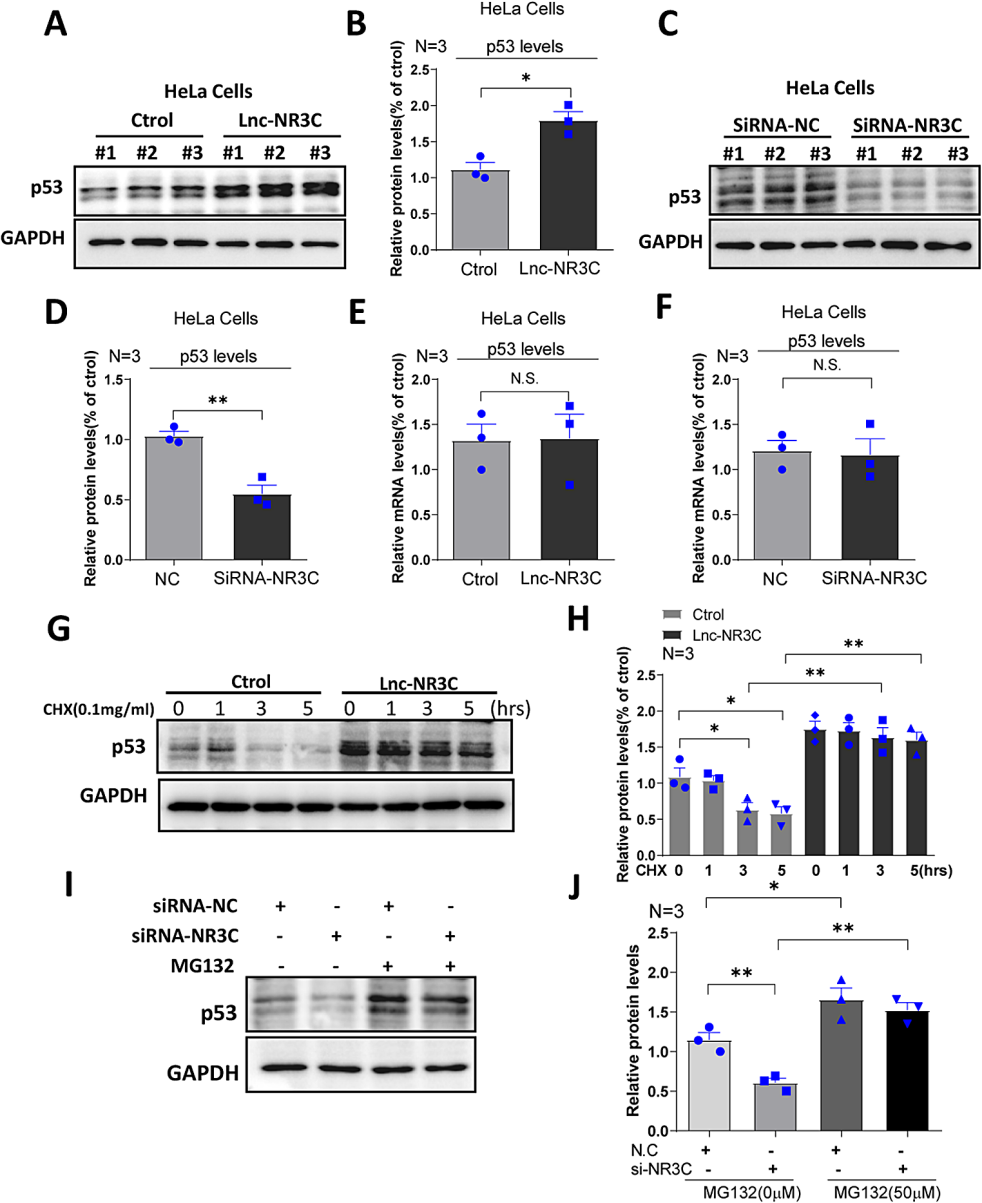
Overexpression of lnc-NR3C increases p53 protein stability. (A-B) Western blot analysis and quantification statistical analysis of the p53 proteins in human HeLa cells with overexpressing lnc-NR3C. (*p < 0.05). (C-D) Western blot analysis and quantification statistical analysis of the p53 proteins in human HeLa cells with knockdown lnc-NR3C. (**p < 0.01). (E-F) qRT–PCR analysis of the p53 in human HeLa cells with overexpressing and knockdown lnc-NR3C. (N.S. no statistical significance. (G-H) Western blot analysis and quantification statistical analysis of the p53 proteins in human HeLa cells treating with cycloheximide (CHX,0.1 mg/ml,1 h,3 and 5 h) after overexpressing lnc-NR3C. (*p < 0.05,**p < 0.01). (I-J) Western blot analysis and quantification statistical analysis of the p53 proteins in human HeLa cells treating with MG132 (50 µM,5 h) after knockdown lnc-NR3C. (*p < 0.05, **p < 0.01) (The concentration of MG132 was incorrectly labelled, and we should correct it to 50 µM in the figure and legends of data J. See the attachment, named Fig. 4)

Several signaling pathways, including the p53 pathway, play critical roles in regulating cell apoptosis. We investigated potential pathways contributing to apoptosis following lnc-NR3C overexpression and found a marked increase in p53 protein levels (Fig. 4A-B and Fig.S2A-B). In contrast, knockdown of lnc-NR3C decreases the p53 protein level (Fig. 4C-D and Fig.S1C-D). However, we did not observe a clear difference at the RNA levels (Fig. 4E-F). This prompted us to explore whether lnc-NR3C influences p53 protein stability. After overexpressing either lnc-NR3C or a vector control in HeLa cells, and treating them with cycloheximide (CHX) to inhibit protein synthesis, we found that p53 levels in lnc-NR3C-overexpressed cells remained stable (Fig. 4G-H), implying that lnc-NR3C overexpression enhances p53 protein stability. Additionally, upon treating lnc-NR3C knockdown HeLa cells with the proteasome inhibitor MG132, we found that p53 protein levels decreased in the absence of lnc-NR3C, but remained stable after MG132 treatment (Fig. 4I-J). This suggests that lnc-NR3C knockdown promotes p53 protein degradation. In summary, our data suggest that lnc-NR3C contributes to the stabilization of p53 protein.

We subsequently explored whether p53 inhibition could mitigate apoptosis induced by lnc-NR3C overexpression. After lnc-NR3C overexpression,we treated cells with pifithrin-α (pft-α), which is a pharmacological inhibitor of p53, and then assessed apoptosis by Hochest/PI staining.Our findings indicated that p53 inhibition, under the condition of lnc-NR3C overexpression, reduced apoptosis following oxidative stress (Fig.S3A-B). These results suggest that the promotion of apoptosis by lnc-NR3C is mediated by p53.

### Lnc-NR3C Overexpression Elevates USP10 Protein Levels

**Fig. 5:**
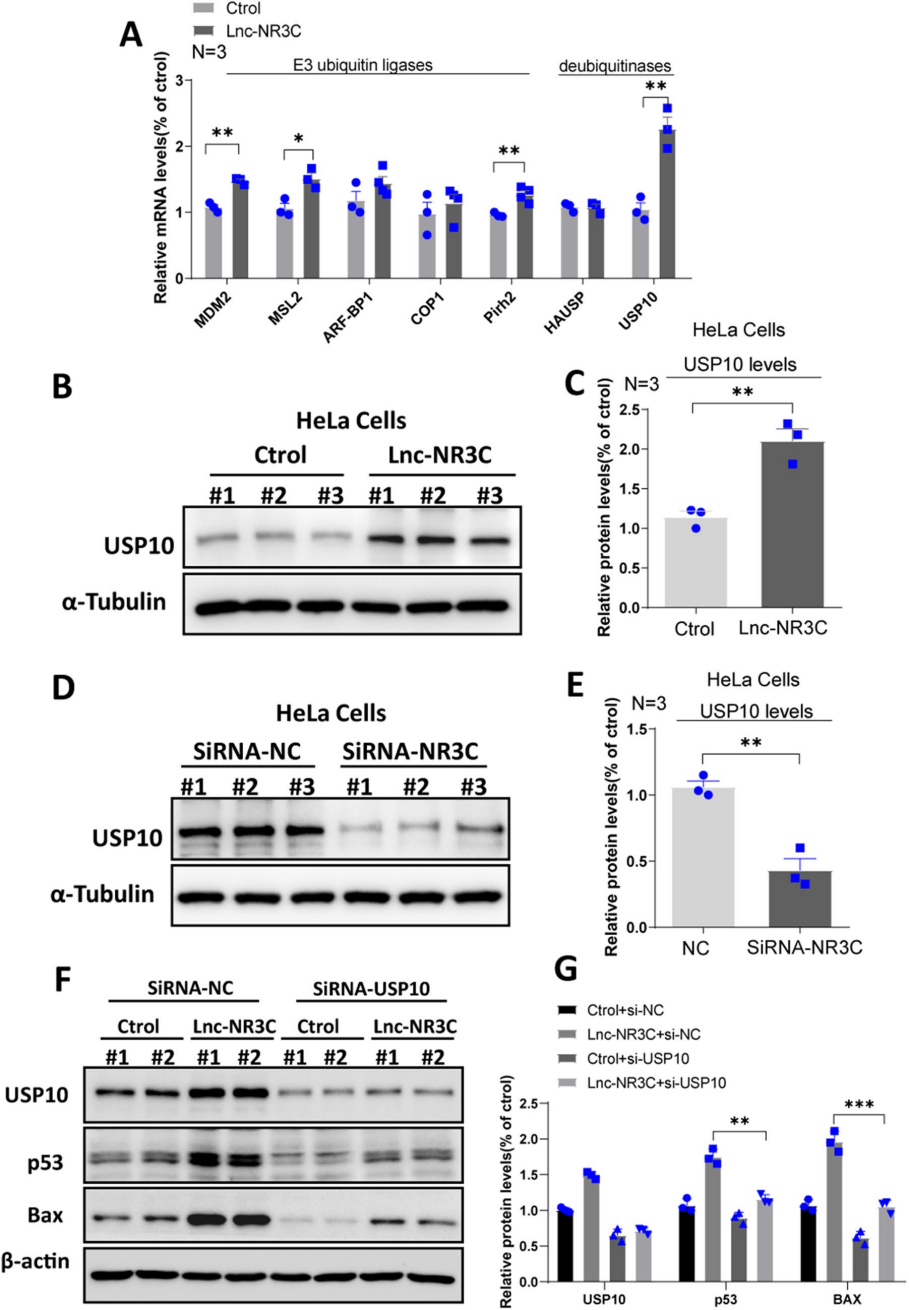
Overexpression of lnc-NR3C increases USP10 protein level. (A) qRT–PCR analysis of E3 ubiquitin ligases and deubiquitinases,which have also been shown to regulate p53 stability, in human HeLa cells with overexpressing lnc-NR3C.(*p < 0.05, **p < 0.01). (B-C)Western blot analysis and quantification statistical analysis of the USP10 proteins in human HeLa cells with overexpressing lnc-NR3C. (**p < 0.01). (D-E)Western blot analysis and quantification statistical analysis of the USP10 proteins in human HeLa cells with knockdown lnc-NR3C. (**p < 0.01). (F-G)Western blot analysis and quantification statistical analysis of the p53 and Bax in human HeLa cells with knockdown USP10 after overexpressing lnc-NR3C. ( **p < 0.01,***p < 0.001)

The protein p53 undergoes degradation through the ubiquitin-proteasome pathway, with its degradation controlled by the level of ubiquitination. E3 ubiquitin ligases and deubiquitinating enzymes (DUBs) govern p53’s stability by modulating its ubiquitination and deubiquitination. We subsequently analyzed the alterations in the E3 ubiquitin ligases and DUBs that regulate p53 activity. Notably, qPCR analysis revealed an elevated RNA level for the E3 ubiquitin ligases MDM2, MSL2, and Pirh2 and the deubiquitinating enzyme USP10 following lnc-NR3C overexpression (Fig. 5A). Given that the increased stability of the p53 protein could be attributed to either heightened E3 ubiquitin ligase activity or diminished DUBs activity, we postulated that lnc-NR3C likely inhibits p53 protein degradation by up-regulating USP10. Confirming this, Western Blot (WB) results demonstrated that lnc-NR3C overexpression augments the USP10 protein level (Fig. 5B-C)while its knockdown reduces it (Fig. 5D-E).

We subsequently explored whether USP10 ablation could mitigate apoptosis induced by lnc-NR3C overexpression. Our findings indicated that USP10 depletion, under the condition of lnc-NR3C overexpression, enhanced cell survival following oxidative stress. WB results further evidenced that lnc-NR3C overexpression escalates p53 and Bax protein levels, whereas USP10 depletion, in the context of lnc-NR3C overexpression, diminishes them (Fig. 5F-G). These findings collectively suggest that lnc-NR3C overexpression enhances the USP10 protein level, leading to p53 up-regulation.

### Lnc-NR3C Functions as a Competing Endogenous RNA by Binding miR-129-5p

**Fig. 6:**
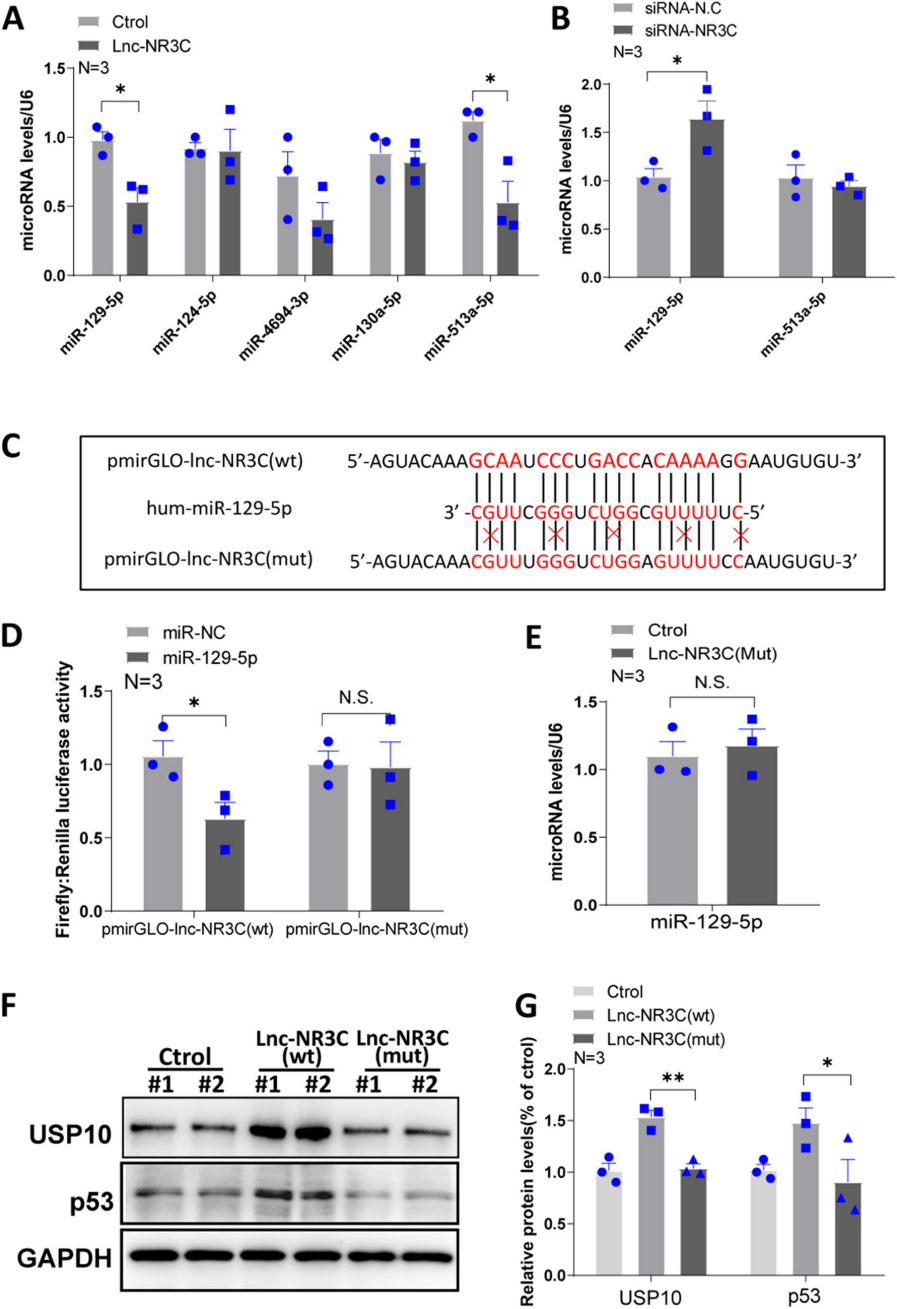
Overexpression of lnc-NR3C increases p53 protein stability. (A) qRT–PCR analysis of potential target miRNAs for lnc-NR3C in human HeLa cells with overexpressing lnc-NR3C. (*p < 0.05). (B) qRT–PCR analysis of potential target miRNAs for lnc-NR3C in human HeLa cells with knockdown lnc-NR3C. (*p < 0.05). (C) Schematic representation of the predicted binding sites for miR-129-5p in lnc-NR3C. (D) Luciferase activity in 293T cells cotransfected with miR-129-5p and luciferase reporters that contained lnc-NR3C (left) or the indicated mutant transcript (right). (*p < 0.05, N.S. no statistical significance). (E) qRT–PCR analysis of miR-129-5p in human HeLa cells with overexpressing mutated lnc-NR3C.(N.S. no statistical significance). (F-G) Western blot analysis and quantification statistical analysis of the USP10 and p53 in human HeLa cells with overexpressing lnc-NR3C and mutated lnc-NR3C. (*p < 0.05,**p < 0.01)

Recently, numerous lnc-RNAs have been identified as competing endogenous RNAs (ceRNAs) that function by binding common microRNAs (miRNAs). To ascertain whether lnc-NR3C operates as a ceRNA, we used multiple microRNA prediction databases like AnnoLnc, Target Scan, miRDB, and DIANA microT-CDS to predict potential binding miRNAs (Fig.S4A-B). Consequently, we narrowed down our selection to miR-129-5p, miR-124-5p, and miR-513a-5p, as the most probable candidates to bind with lnc-NR3C. Moreover, qRT-PCR results showed significant downregulation of miR-129-5p and miR-513a-5p following lnc-NR3C overexpression in HeLa cells (Fig. 6A), while only miR-129-5p was significantly upregulated post lnc-NR3C silencing (Fig. 6B).

To validate the interaction between lnc-NR3C and miR-129-5p, we conducted dual luciferase reporter assays based on the predicted interaction site from RNA22 and RNAhybrid 2.2 databases (Fig. 6C). MiR-129-5p overexpression resulted in reduced luciferase activity of the lnc-NR3C reporter vector (Fig. 6C). We also engineered luciferase reporters with a mutated lnc-NR3C sequence that contained potential miR-129-5p binding sites. After transfection into 293T cells, miR-129-5p overexpression failed to reduce the luciferase activity of the mutated lnc-NR3C vector (Fig. 6D). Moreover, when a vector containing a mutated lnc-NR3C sequence at the miR-129-5p binding site was introduced into HeLa cells, there was no significant change in miR-129-5p levels (Fig. 6E). WB results showed that while lnc-NR3C overexpression increased USP10 and p53 protein levels, overexpression of the mutated lnc-NR3C did not. These findings suggest that the increase in USP10 and p53 protein levels via lnc-NR3C overexpression is reliant on its interaction with miR-129-5p. In conclusion, it appears that lnc-NR3C may serve as a ceRNA for miR-129-5p.

### MiR-129-5p Inhibits USP10 Expression

**Fig. 7:**
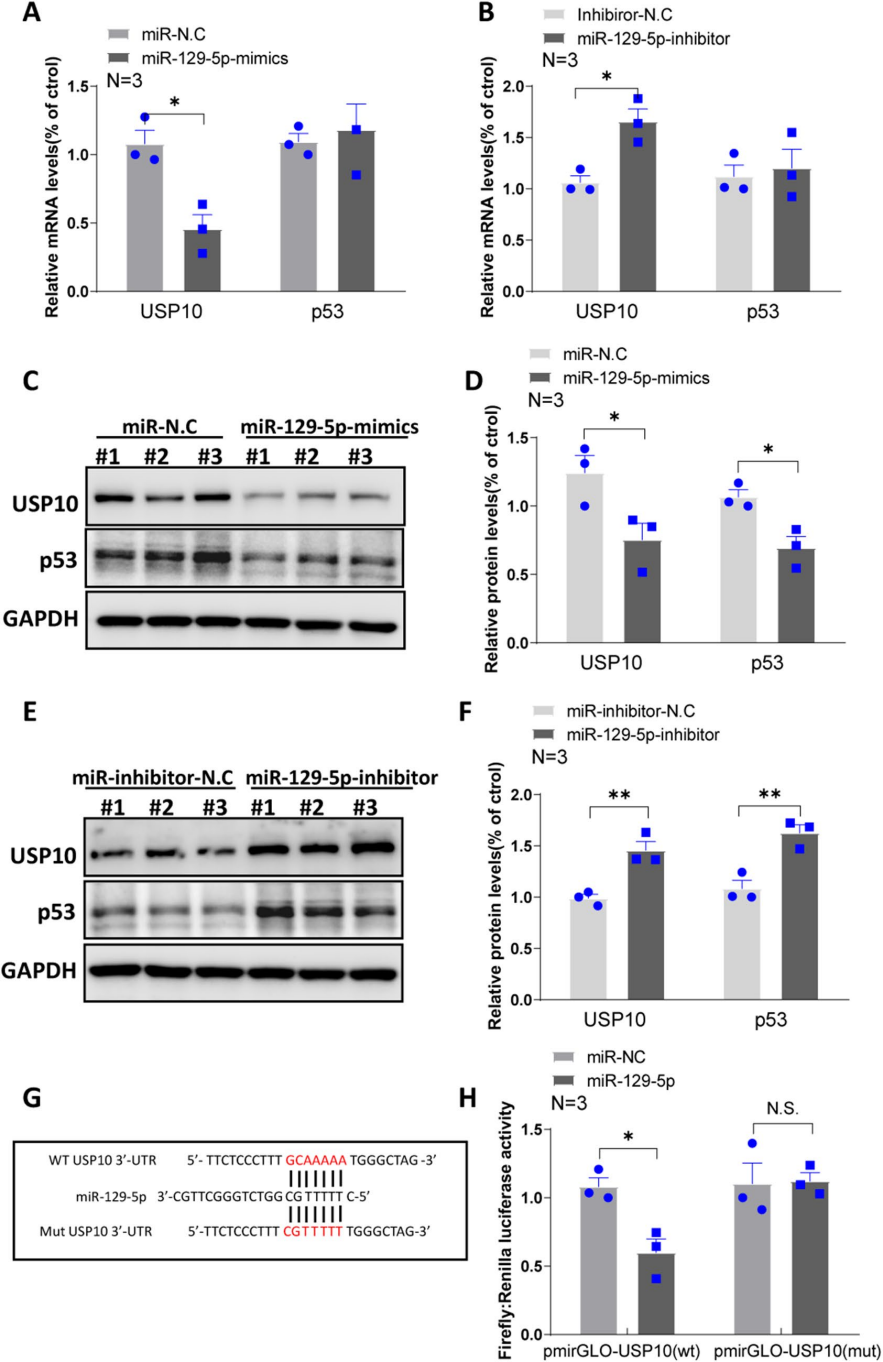
miR-129-5p inhibits the expression of USP10. (A-B) qRT–PCR analysis of the USP10 and p53 in human HeLa cells with overexpressing and inhibiting miR-129-5p. (*p < 0.05). (C-D) Western blot analysis and quantification statistical analysis of the USP10 and p53 in human HeLa cells with overexpressing miR-129-5p. (*p < 0.05). (E-F) Western blot analysis and quantification statistical analysis of the USP10 and p53 in human HeLa cells with inhibiting miR-129-5p. (*p < 0.05,**p < 0.01). (G) Schematic representation of the predicted binding sites for miR-129-5p in USP10 3’-UTR. (H) Luciferase activity in 293T cells cotransfected with miR-129-5p and luciferase reporters that contained USP10 3’-UTR (left) or the indicated mutant transcript (right). (*p < 0.05, N.S. no statistical significance)

To identify genes sharing miR-129-5p’s regulatory role with lnc-NR3C, we predicted hundreds of miR-129-5p target genes using Targetscan. Notably, USP10 emerged as a potential target gene for miR-129-5p. To further verify miR-129-5p’s regulatory impact on USP10, we examined USP10’s RNA and protein levels in HeLa cells following miR-129-5p overexpression or inhibition. The qPCR results revealed a significant decrease in USP10 RNA levels upon miR-129-5p overexpression, while its inhibition led to an increase (Fig. 7A-B). Correspondingly, WB results showed a similar pattern on the protein level (Fig. 7C-F). To confirm USP10 as a direct target of miR-129-5p, we performed a dual-luciferase reporter assay using a vector containing the USP10 3’UTR sequence. Here, miR-129-5p overexpression significantly reduced luciferase activity, while the mutant vector was unaffected (Fig. 7G-H).

In summary, miR-129-5p appears to inhibit USP10 expression at both the RNA and protein levels. This inhibition, combined with the potential ceRNA activity of lnc-NR3C, suggests a complex regulatory network underpinning p53 stability. Our findings highlight the importance of understanding the multifaceted interplay between lnc-NR3C, miR-129-5p, and USP10 in the regulation of p53, which could provide novel insights into p53-related pathologies and therapies.

## Discussion

The pathogenesis of Amyotrophic Lateral Sclerosis (ALS) remains intricate and not fully understood. Recent research has highlighted the pivotal role of RNA metabolism alterations in ALS [34], with a particular emphasis on long non-coding RNAs (lncRNAs) [31, 35–37]. In our previous study, we identified 30 lncRNAs with differential expression from the peripheral blood leukocytes of sporadic ALS (SALS) patients and validated 13 of them, including lnc-NR3C. The current investigation delves deeper into the expression and function of lnc-NR3C. Our findings suggest that a reduction in lnc-NR3C exerts a protective effect against in vitro induced apoptosis in HeLa and SHSY5Y cells. This reduction down-regulates the protein level of p53, while its overexpression up-regulates the same protein level to promote apoptosis. Furthermore, our study demonstrates that lnc-NR3C, acting as a competing endogenous RNA (ceRNA), upregulates USP10 expression by sequestering miR-129-5p, thereby augmenting the stability of the p53 protein and promoting apoptosis. Consequently, we propose that decreased expression levels of lnc-NR3C could potentially confer a protective role in ALS patients.

Currently, ALS lacks effective cures, with only three treatment options approved in the United States: Riluzole, Edaravone, and Dextromethorphan [38, 39]. This situation is partly due to the wide diversity of human genetic backgrounds, leading to some therapeutic strategies proving effective in ALS mouse models but largely ineffective in ALS patients. LncRNAs, which are poorly conserved and play significant roles in the regulation of numerous physiological processes and diseases, could be potential therapeutic targets for ALS. In fact, several studies have underscored the role of lncRNAs in ALS progression, such as lncRNA-NEAT1_2 [31]. Our study reports that lnc-NR3C, a newly identified lncRNA which is poorly conserved and not expressed in mouse tissues, is involved in p53-mediated apoptosis, and lnc-NR3C knockdown cells exhibit resistance to apoptosis. Given that motor neuron death is a primary characteristic of ALS, we believe that lnc-NR3C could be a potential therapeutic target for ALS.Lnc-NR3C is poorly conserved so that our study lacked in vivo confirmation and our results should be confirmed in vivo with future animal experiments. Recently we established a resource of about 30 fully characterized iPSC lines, including ALS-iPSC lines and PD-iPSC lines [40], in the following study we will use the iPSC model to verify the conclusions of this study.

The p53 signaling pathway is crucial in cellular function and is strongly associated with ALS. Some findings support p53 activation in ALS [16], particularly with C9orf72 repeat expansions [23]. Recently, a study conducted by Oliver J and colleagues also discovered strong and significant activation of p53 in TARDBP and sporadic subgroups [17]. Therefore, targeting p53 signals a potentially promising approach for ALS prevention and therapy. In our study, we have newly uncovered a method to control p53 activity in human cells, where lnc-NR3C activates p53 signaling by increasing USP10 expression. Consequently, the intrinsic regulator lnc-NR3C should be considered when targeting p53 in ALS treatment.

The lncRNA-miRNA-mRNA competitive endogenous RNA regulatory network is a common molecular regulatory mechanism for lncRNA. In most cases, lncRNA can act as natural miRNA sponges to adsorb and inhibit the function of miRNAs. Our research uncovered that lnc-NR3C sequesters to inhibit the function of miR-129-5p. While we reported a decrease in lnc-NR3C in the peripheral blood cells of sporadic ALS patients, a recent study has shown an increase in miR-129-5p in the same cells and in different models of SOD1-linked ALS [41]. These findings suggest a close relationship between lnc-NR3C and miR-129-5p in ALS. As one lncRNA can regulate multiple miRNAs simultaneously, and one miRNA can regulate numerous genes, we discovered that lnc-NR3C also bound with another miRNA, indicating that lnc-NR3C might possess other functions.

**Fig. 8:**
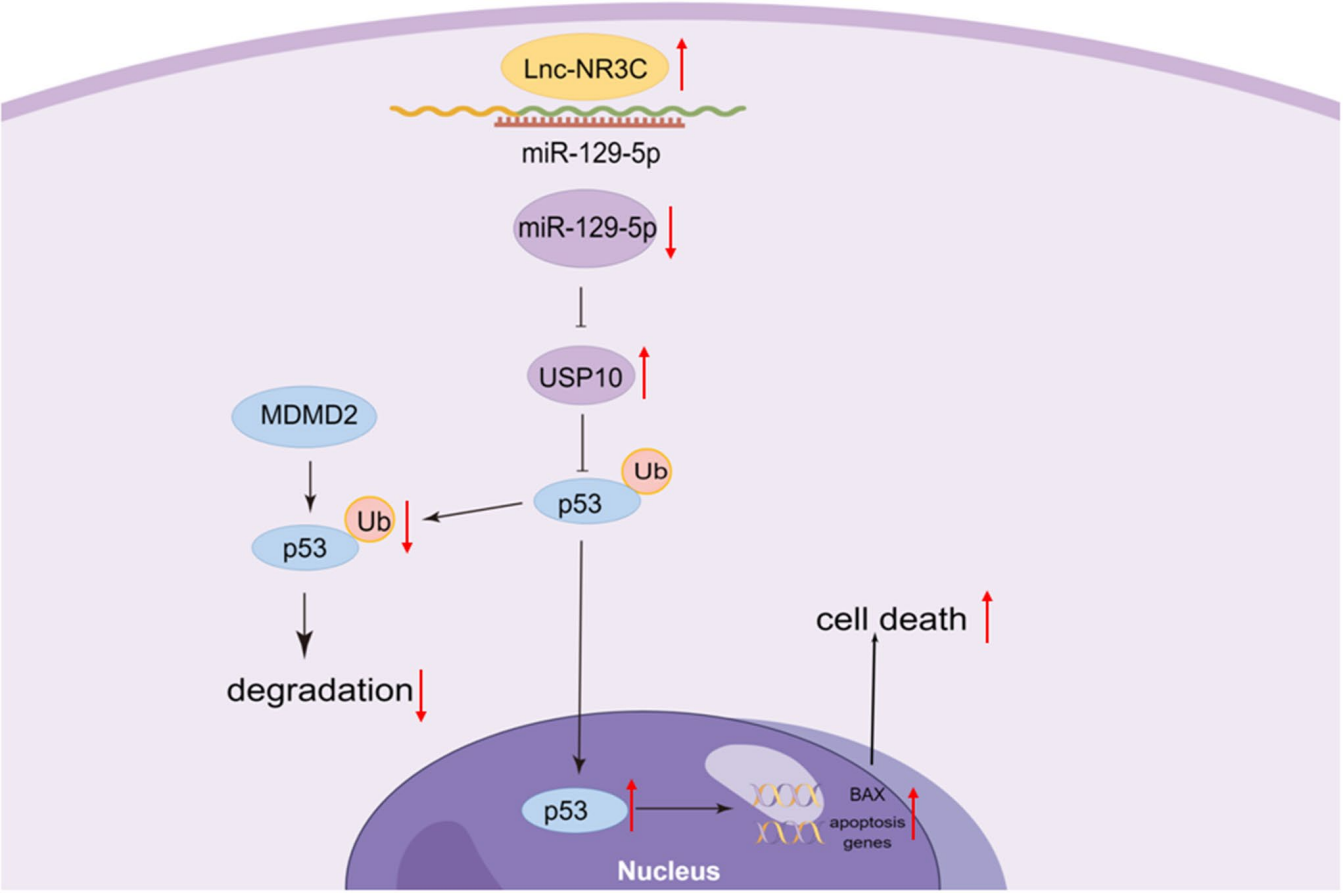
Model. The long non-coding RNA NR3C2-8:1(lnc-NR3C) promotes p53-mediated apoptosis through the miR-129-5p/USP10 Axis

In conclusion, we characterized the expression of lnc-NR3C in the human brain and found that lnc-NR3C was localized in the cell cytoplasm. lnc-NR3C was involved in cell survival and promoted apoptosis via p53 signaling. As a ceRNA, lnc-NR3C upregulated the activity of the USP10/p53 axis by sponging miR-129-5p, which inhibited the expression of USP10 (Fig. 8). These results provide novel insights into the molecular pathogenesis of ALS and suggest potential novel therapeutic targets.

## Materials and methods

### Cell Cultures and Treatments

SHSY5Y cells (human neuroblastoma cells), HeLa cells, and 293T cells were procured from the Cell Bank of the Chinese Academy of Sciences. Cells were cultured in DMEM medium (for HeLa and 293T) or DMEM/F12 medium (for SHSY5Y) supplemented with 10% foetal bovine serum (FBS), 2mM L-glutamine, and 1% penicillin/streptomycin. For in vitro experiments, cells were treated with 200µM H_2_O_2_ or 25µM rotenone (from MCE) for a duration of 24 h, followed by further analyses.

### Basic Information on Lnc-NR3C2-8:1

The LNCipedia transcript ID for lnc-NR3C is lnc-NR3C2-8:1, and its location (hg38) is on chr4:147638681–147,639,293. The strand is negative, the class is intronic, and the Sequence Ontology term is sense_intronic_ncRNA. The transcript size is 401 bp, with two exons. Its sources include NONCODE v4, and its alternative transcript names include NONHSAT098688. The lnc-NR3C2-8:1 transcript was not conserved, and there was no expression in mouse tissues or cells.

### Fluorescence in Situ Hybridization

Fluorescence in situ hybridization was performed as described by the manufacturer’s instructions (GenePharma). Briefly, SHSY5Y and HeLa cells were fixed with 4% RNase-free PFA and then hybridized with a biotin-labelled probe corresponding to lnc-NR3C. The probe sequence of lnc-NR3C is GT + TAGAGGTGT + TCAAATACGG AGTC. Lastly, DAPI was used for nuclear staining, and images were captured using a fluorescence microscope.

### Patients and Clinical Samples

Peripheral blood samples from healthy individuals were procured from the Department of Neurology, West China Hospital of Sichuan University. Brain tissue samples were collected from patients diagnosed with glioma who underwent surgery at the Department of Neurosurgery of the same hospital. Informed consent was obtained from all participants during the course of the study. The study received approval from the institutional ethics board of our hospital.

### RNA Extraction and Quantitative Real-Time PCR Assays

Total RNAs and miRNAs were extracted using the Trizol method (Invitrogen). Subsequently, reverse transcription was carried out using a reverse transcription kit (Takara, #2641A). Then, quantitative real-time PCR assays (qPCR) was performed using a Takara qPCR kit (Takara, #RR82LR). The qPCR primer sequences are shown in supplemental Table 1.

For miRNA detection, reverse transcription was performed, and miRNA expression was measured using the All-in-One™ miRNA qRT-PCR Detection Kit (Genecopoaie, Lot#QP015) according to the user manual. MicroRNA qPCR primers were purchase from Genecopoaie as follow: has-miR-129-5p-HmiRQP0137, has-miR-124-5p- HmiRQP0073, has-miR-4694-3p-HmiRQP2318, has-miR-513a-5p-HmiRQP0566, has-miR-130a-5p-HmiRQP0157.

### Transient Transfection

Transient transfection was executed with Polyplus jetPRIME (Polyplus Transfection, #114 − 15) following the manufacturer’s instructions. Cells were seeded in a 6-well plate 24 h prior to transfection, and each well was transfected with 2 µg of the GV146-lnc-NR3C plasmid (for overexpressing lnc-NR3C) (Genechem). The siRNA (for knockdown of lnc-NR3C), along with miRNA-129-5p mimics and inhibitors (GenePharma), were introduced into the cells at a final concentration of 50nM. The cells were harvested 48 h post-transfection.

To exclude any siRNA off-target effect, three sequences were used. The lnc-NR3C siRNA target sequences were as follows: siRNA-1, 5′-GGAAUAUAAAG GAGGUAAUTT′; siRNA-2, 5′-GCCAGUGGAACUCUUAAGATT-3′; siRNA-3, 5′-GGUAUACGUUUGUAAAUCUTT-3′,and NC, 5′-UUCUCCGAACGUGUCACG UTT-3′. Each siRNA was transfected into HeLa or SHSY5Y cells, and the qPCR results shown that each siRNA reduced the mRNA level of lnc-NR3C (Fig.S1A-B). In our following study,these three kinds of siRNAs were mixed (siRNA pool) to achieve the interference effect while eliminating the off-target effect by reducing the use of each kind of siRNA.

### Cell Viability Analysis

The cell viability was determined using a Dojindo Laboratories cell-counting kit-8, in accordance with the provided manufacturer’s instructions. Approximately 1.0 × 10³ cells per well were seeded into 96-well plates and were allowed to culture overnight. Following this, the cells were transfected with either an lnc-NR3C overexpression plasmid or an siRNA knockdown. Post-transfection (24 h later), the cells were subjected to the specified drug treatments. At each designated time point, a volume of 10 µl of CCK8 solution was added and allowed to incubate. The staining intensity, indicative of cell viability, was quantified by measuring the absorbance at 450 nm.

### Western Blot

Western blotting was performed according to standard procedures as reported before [42]. To extract protein from cultured cells was sonicated in lysis buffer (2% SDS with proteinase inhibitors and phosphatase inhibitor). The protein concentration of each extract was measured using the BCA Protein Assay kit (Thermo Scientific Pierce). Equal amounts of protein from each extract were loaded into each lane of a gel and separated by SDS-PAGE. The proteins were transferred onto PVDF membranes using standard procedures. The membranes were then blocked with 5% nonfat dry milk in TBST (TBS with 0.1% Tween20, pH 7.6) for 1 h at room temperature (RT) and incubated overnight with respected primary antibody at 4 °C.After 3 times washes with TBST at RT for 10 min, the membranes were incubated 1 h with a 1:10,000 dilution of appropriate secondary antibodies diluted in TBST at RT. The membranes were washed another 3 times with TBST at RT for 10 min, proteins were then detected with ECL reagent (Thermo Scientific Pierce).The antibodies employed in this study were as follows: Bax (#2772, CST), Bcl-2 (#15,071, CST), Cleaved-PARP1 (ET1608-10, HUABIO), alpha-Tubulin (#3873, CST), p53 (10442-1-AP, Proteintech), USP10 (19374-1-AP, Proteintech), MDM2 (19058-1-AP, Proteintech), HAUSP (66514-1-Ig, Proteintech), and GAPDH (60004-1-Ig, Proteintech).

### Bioinformatics Analysis

The target miRNA of lnc-NR3C was identified using four distinct online databases: miRDB, microRNA.org, TargetScan, and Diana Tools.

### Luciferase Reporter Assay

The luciferase constructs were generated using a luciferase reporter vector, specifically the pmirGLO dual-luciferase miRNA target expression vector from GenePharma. Then, pmirGLO, pmirGLO-lnc-NR3C, or pmirGLO-lnc-NR3C-mut (miR-129-5p) was cotransfected with a miR-129-5p mimic in 293T cells. After 24 h of transfection, the relative luciferase activity was normalized to renilla luciferase activity and quantified using a fluorescence microplate reader, as per the manufacturer’s protocol. Likewise, analogous experiments were conducted to ascertain if USP10 was a direct target of miR-129-5p, as described above.

### Statistical Analysis

The statistical analysis was conducted using GraphPad Prism 8 software. Significant statistical differences between the two groups were determined by a two-tailed unpaired Student’s t-test. For comparisons of three or more groups, a one-way ANOVA analysis was performed. A p-value less than 0.05 was deemed significant: p-value < 0.05 (*), p-value < 0.01 (**), p-value < 0.001 (***), N.S.(no statistical significance).

## Electronic Supplementary Material

Below is the link to the electronic supplementary material.

Supplementary Material 1

## Data Availability

The data that support the findings of this study are available from the corresponding author upon reasonable request.
