# Supplementary material for: The Long Non-Coding RNA NR3C2-8:1 Promotes p53-Mediated Apoptosis through the miR-129-5p/USP10 Axis in Amyotrophic Lateral Sclerosis
Source: Mol Neurobiol. 2024 Feb 23;61(10):7466–80. doi: 10.1007/s12035-024-04059-x (PMC11415439; doi:10.1007/s12035-024-04059-x)

**Supplementary data**

**Figure S1. Verification of transfection efficacy of lnc-NR3C siRNA.**

(A)QRT–PCR analysis of the lnc-NR3C in human HeLa cells with knockdown lnc-NR3C by siRNA-1,siRNA-2,siRNA-3 and siRNA-mix.(*p < 0.05,**p < 0.01)

(B) QRT–PCR analysis of the lnc-NR3C in human SHSY5Y cells with knockdown lnc-NR3C by siRNA-1,siRNA-2,siRNA-3 and siRNA-mix. (**p < 0.01,***p < 0.001)

**
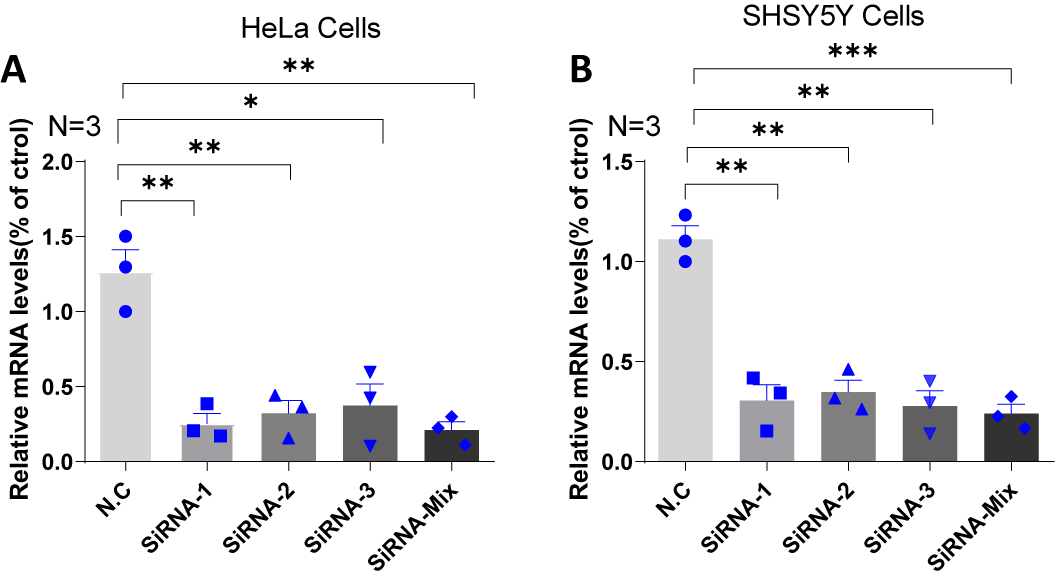
**

**Figure S2. Overexpression of lnc-NR3C increases p53 protein level.**

(A-B) Western blot analysis and quantification statistical analysis of the p53 proteins in human SHSY5Y cells with overexpressing lnc-NR3C. (**p < 0.01)

(C-D) Western blot analysis and quantification statistical analysis of the p53 proteins in human SHSY5Y cells with knockdown lnc-NR3C. (**p < 0.01)

**
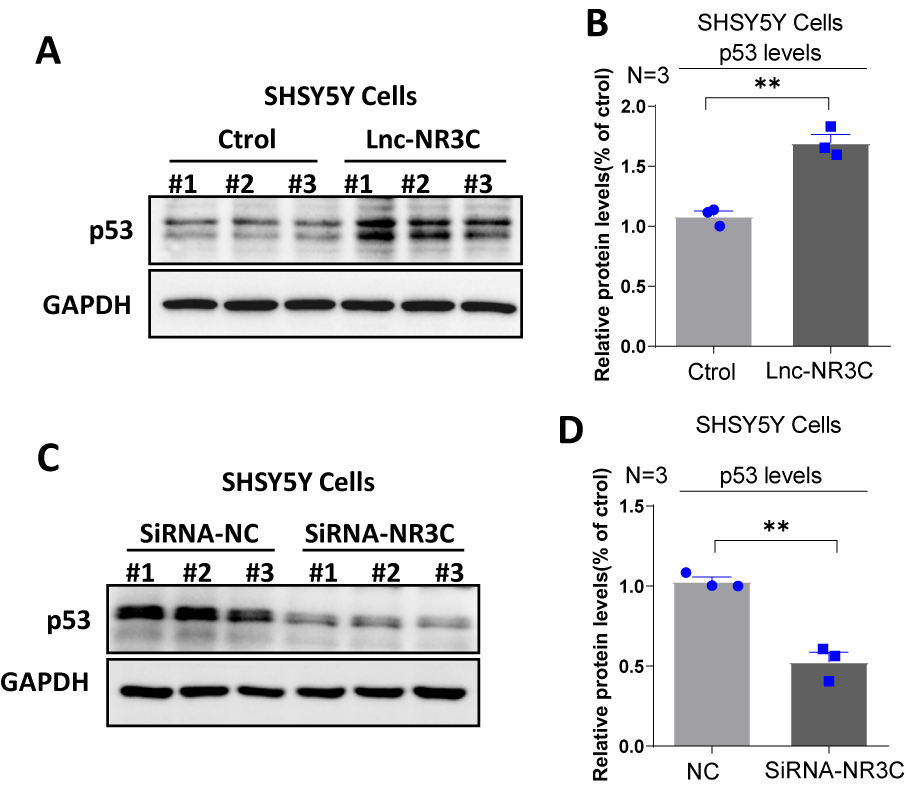
**

**Figure S3. p53 inhibition could mitigate apoptosis induced by lnc-NR3C overexpression.**

(A-B) After overexpressing lnc-NR3C cell apoptosis were assessed by Hochest/PI staining in the presence of H_2_O_2_(200μM,24h) and Pft-α(20μM,24h).Cells were costained with Hoechst (blue) and PI (red), and visualized by fluorescence microscope (A).The total cell death is statistically analysed(B). (*p < 0.05,**p < 0.01).

**
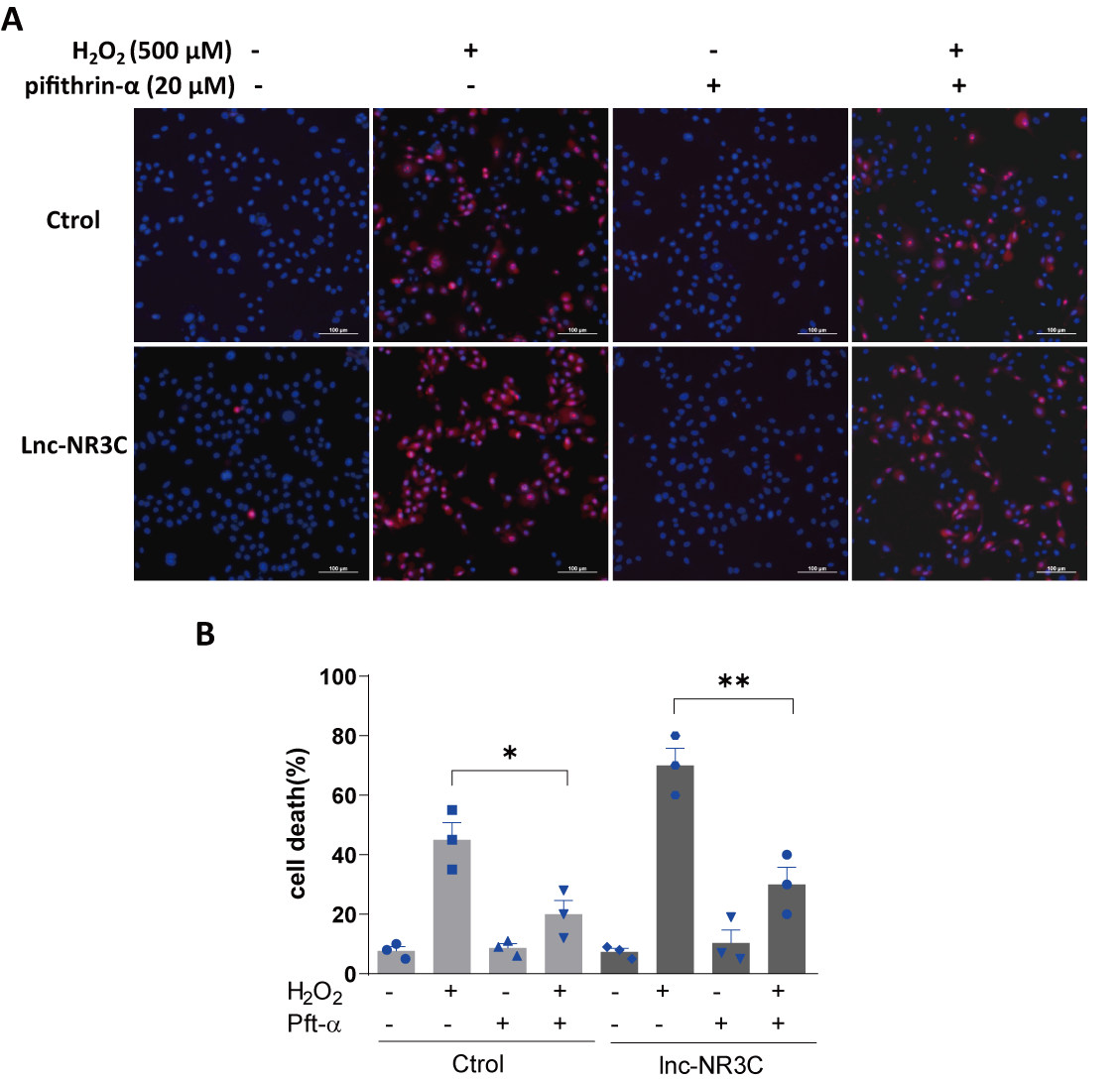
**

**Figure S4. Predict potential** **binding miRNAs with lnc-NR3C**

(A)Prediction results of lnc-NR3C binding miRNAs in multiple microRNA prediction databases were shown by a Venn diagram.

(B)Some candidate miRNAs were selected from both miRNA prediction datasets.

**
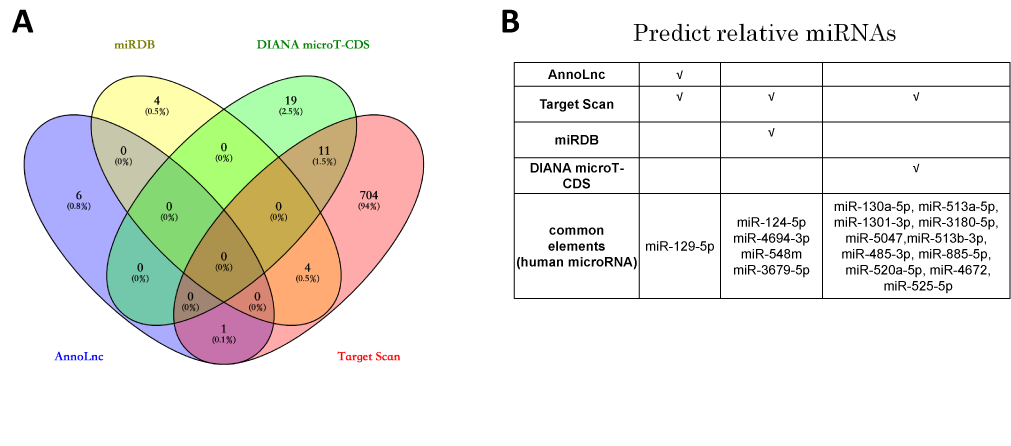
**

**Supplemental table 1. QPCR primers used in this study.**


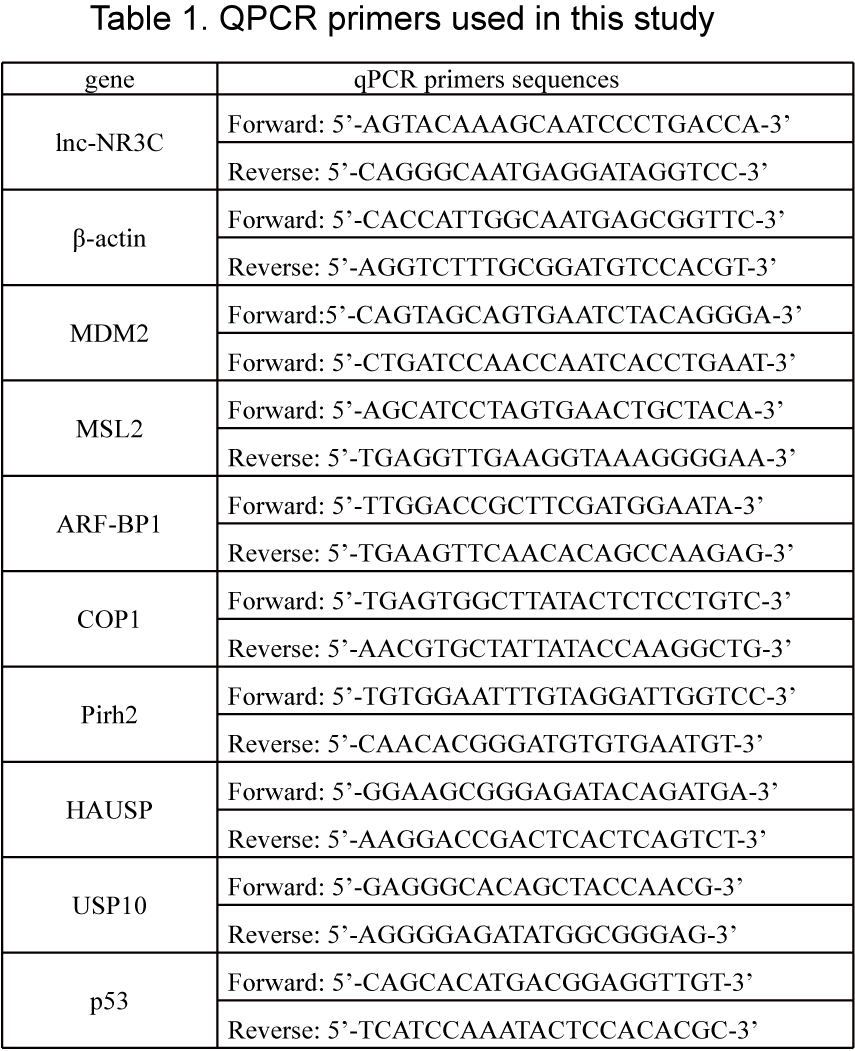

Supplement: Supplementary file 1 — Supplementary Material 1 [file 12035_2024_4059_MOESM1_ESM.docx]
